# The functional effects of African lions on co-occurring carnivores differ across species pairs and with changes in resource availability and lion abundance

**DOI:** 10.1007/s00442-025-05855-5

**Published:** 2026-01-08

**Authors:** Kristoffer T. Everatt, Leah Andresen, Jennifer F. Moore, James E. Hines, Graham I. H. Kerley

**Affiliations:** 1 Centre for African Conservation Ecology, Department of Zoology, Nelson Mandela University, Gqeberha, 6031 South Africa; 2 Panthera, New York, NY USA; 3 Moore Ecological Analysis and Management, LLC, St. Petersburg, FL USA; 4 Eastern Ecological Science Center, USGS, Laurel, MD USA; 5 Box 276, Hagensborg, BC V0T 1H0 Canada

**Keywords:** Cheetah, Leopard, African wild dog, Competition, Two-species occupancy, Tracking, Limpopo, Kruger

## Abstract

Apex carnivores are known to regulate ecosystem structure and function, including via interactions with syntopic, competitively inferior carnivores. These effects may be dependent on relative carnivore density and resource availability or productivity. We investigated the functional effect of African lions as an apex carnivore on the presence of co-occurring large carnivore species across two adjoining National Parks that contrast in relative densities of carnivores and prey. We employed two-species occupancy models from track data to test statistical interactions between lions and the other syntopic large carnivore species, while accounting for each species’ habitat selection. We further investigated the influence of anthropogenic and natural variables on these co-occurrence dynamics. Our models revealed that the occurrence of each carnivore species was best predicted by access to their own key resources. We also found significant statistical interactions between lions and cheetahs, lions and leopards, and lions and spotted hyenas in resource-rich landscapes. Finally, we found limited support for the competition exclusion hypothesis between most species, with the exception of lion-African wild dog co-occurrence patterns. Species’ co-occurrence dynamics were all influenced by resource availability, with lion-leopard and lion-cheetah co-occurrence decreasing strongly with increasing resource availability. Most species co-occurrence declined with increasing occurrence of lions. The patterns revealed by this study improves predictions of how changes in resource availability and carnivore occurrence could impact carnivore community dynamics and the functional role of apex carnivores.

**Supplementary Information:**

The online version contains supplementary material available at 10.1007/s00442-025-05855-5.

## Introduction

Carnivore communities are known to be structured by competition, including predation, harassment, displacement, and kleptoparatisism (Périquet et al. 2015; Cornhill et al. 2023). The relative susceptibility of carnivore species to intra-guild influences may differ according to each species’ hunting strategies, physical prowess, physiological requirements, and sociality (Vanak et al. 2013; Périquet et al. 2015; Haswell et al. 2017), as well as the diversity and redundancy within the carnivore guild (Suraci et al. 2017). In addition, relative primary productivity (Mallory et al. 2019), temporal or seasonal shifts in resource availability (Stoessel et al. 2019), and prey diversity (Brashares et al. 2010) all influence the strength of intra-guild interactions.

Co-existence between carnivore species is, however, widespread and facilitated through dietary, spatial, and temporal niche partitioning or avoidance (Vanak et al. 2013; Cornhill et al. 2023). Such evolutionary processes have resulted in the existence of diverse carnivore assemblages, which for example can still be found in the few remaining intact African savanna ecosystems.

African lions *Panthera leo*, the largest and most competitively dominant African carnivore, are known to kleptoparasitize from, displace and kill syntopic cheetah *Acinonyx jubatus*, leopard *Panthera pardus*, spotted hyenas *Crocuta crocuta*, and African wild dogs *Lycaon pictus* (Palomares and Caro 1999; Groom et al. 2017; Cornhill et al. 2022). Consequentially, each of these species have been shown to display various forms of behavioral avoidance of lions (Balme et al. 2017; Groom et al. 2017; Lehmann et al. 2017; Cornhill et al. 2022).

The conservation status of many of the world’s apex carnivores has been rapidly declining with increasing anthropogenic pressures, leaving intact carnivore guilds becoming ever more infrequent (Ripple et al. 2014; Fernández-Sepúlveda and Martín 2022). Even within intact guilds, anthropogenic pressures often limit individual carnivore species densities (Ripple et al. 2014) and impact carnivore behavior (Barker et al. 2023; Everatt et al. 2023). Anthropogenic pressures may impact the ability of apex carnivores to structure guilds and thus fulfill their ecological roles (Atkins et al. 2019). It is therefore increasingly important to consider top-down anthropogenic influences when examining carnivore community dynamics (Kuijper et al. 2016; Everatt et al. 2016).

Here we investigate a functional effect of lions in structuring an intact large carnivore guild through competition, and if such an effect was influenced by relative lion occupancy and resource availability, or lack thereof as a result of anthropogenic pressures. We considered co-occurrence patterns between species pairs to test the following hypotheses: (1) that in accordance with competitive exclusion theory, the four sub-ordinant sympatric large carnivores (cheetah, leopard, spotted hyena, and African wild dog) would avoid competitively superior lions, or the alternate hypothesis (2) that in accordance with niche partitioning, these carnivores would display high co-occurrence. If there is evidence of limitation of sympatric carnivores by lions, we then also considered the following three hypotheses; (3) the degree of lion-sympatric carnivore co-occurrences would be related to the relative abundance of lions, with lower lion occupancy having limited influence on sympatric carnivores, or (4) that the degree of lion-sympatric carnivore co-occurrence would be related to the availability of resources and thus the opportunity for niche partitioning between species, and finally, (5) that these species’ co-occurrence are independent of lion occupancy or resource availability (See Table 1).

**Table 1:** Independent variables expected to describe lion, cheetah, leopard, spotted hyena, and African wild dog habitat use, their assumed biological relevance to carnivore occurrence, metric recorded, and data source

Independent variable	Biological relevance to carnivore occurrence	Metric recorded	Source
Buffalo (occupancy)	Preferred prey for lions and spotted hyena^1,2^	Detection/non-detection on each 1 km sampling occasion (hereafter 1 km)	This survey
Impala (occupancy)	Preferred cheetah, leopard, and African wild dog prey^1^ alternate prey for lions and spotted hyena^1,2^	Detection/non-detection on each 1 km	This survey
Kudu (occupancy)	Preferred prey for wild dogs, alternate prey for lions, cheetah, and spotted hyena^1,2^	Detection/non-detection on each 1 km	This survey
Cattle (occupancy)	Persecuting and possible prey, displacement of carnivore^3,4^	Detection/non-detection on each 1 km	This survey
Poaching (occupancy)	Direct persecution and/or depletion of prey, persecution or displacement of carnivore^5^	Detection/non-detection on each 1 km	This survey
Settlements/agriculture (distance from)	Direct persecution and/or depletion of prey^6^	Euclidean distance of 1 km from settlement (30 × 30 m) pixels	Landcover raster^7^
Protected areas (distance from)	Higher protection in Kruger^5^	Euclidean distance of 1 km from center of Kruger	Landcover raster^7^
Water	Habitat feature that may influence prey availability	Number of (30 × 30 m) pixels overlapping 1 km	Landcover raster^7^
Pans	Habitat feature that may influence hunting success or movements^2^	Number of (30 × 30 m) pixels overlapping 1 km	Landcover raster^7^
Grasslands	Habitat feature that may influence hunting success or movements^2^	Number of (30 × 30 m) pixels overlapping 1 km	Landcover raster^7^
Forests	Habitat feature that may influence hunting success or movements^2^	Number of (30 × 30 m) pixels overlapping 1 km	Landcover raster^7^
Bushlands	Habitat feature that may influence hunting success or movements^2^	Number of (30 × 30 m) pixels overlapping 1 km	Landcover raster^7^
Thickets	Habitat feature that may influence hunting success or movements^2^	Number of (30 × 30 m) pixels overlapping 1 km	Landcover raster^7^

^1^Hayward et al. 2007; ^2^ Funston et al. 2001; ^3^ Forbes et al. 2024; ^4^ Everatt et al. 2023; ^5^Everatt et al. 2019b; ^6^Everatt et al. 2019a; ^7^Extracted from Imagine Image raster “GLTF Landcov projected in WGS_1984_UTM_Zone_36S (www.peaceparks.co.za) in ArcGIS 10.1 (www.esri.com)

## Methods

### Study area and population

Data were collected from two contiguous sampling areas: the northern portion of Kruger National Park (hereafter “Kruger”) in South Africa and the adjoining Limpopo National Park (hereafter “Limpopo”) in Mozambique, each approximately 10,000 km^2^. The international border fence separating the two has been made permeable to wildlife movements as part of these parks’ inclusion in the Greater Limpopo Conservation Area (Fig. S.1). Vegetation across the region can be characterized as mixed savanna woodlands and grasslands (Gertenbach 1983; Stalmans et al. 2004). Important differences between the two sampling areas are the types of anthropogenic pressures characterized in each park. Kruger is a well-protected area with high non-consumptive photographic tourist visitation, while Limpopo is inhabited by approximately 6000 residents grazing approximately 20,000 cattle and is characterized by relatively high amounts of wildlife poaching and pastoralism, including the persecution of large carnivores in response to actual or perceived livestock depredation (Everatt et al. 2019a). This difference translates to high persecution of carnivores in Limpopo and low persecution of carnivores in Kruger (Everatt et al. 2019b). At the time of the study, lion densities were over 6 × greater in northern Kruger than in Limpopo, with estimated densities of 6.1/100 km^2^ in northern Kruger (Ferreira and Funston 2010) and 0.99/100 km^2^ in Limpopo (Everatt et al. 2014). Spotted hyena densities were 15 × greater in northern Kruger than in Limpopo, with densities of 15.33/100 km^2^ in northern Kruger (Ferreira and Funston 2016) and 1.0/100 km^2^ in Limpopo (Everatt et al. 2019b). There are limited data on leopard, cheetah, and African wild dog densities across the study area. Extrapolations however suggest that there are 1000 leopards in northern Kruger (Maputla et al. 2013) and only 87 leopards in Limpopo (Forbes et al. 2025). Cheetah and African wild dog both range across northern Kruger and Limpopo with comparable densities. There are a recorded minimum of 42 cheetahs in northern Kruger (Marnewick et al. 2014) and 35 in Limpopo (Everatt unpublished), and approximately 24 African wild dogs in northern Kruger (Marnewick et al. 2014) and 20 African wild dogs in Limpopo (Everatt, unpublished).

### Study design

We employed the use of two-species occupancy models to test for statistical interactions between species pairs to provide inferences on the role of competition in community structure and species’ co-occurrence patterns (see Robinson et al. 2014; Kafley et al. 2019).

**Fig. 1:**
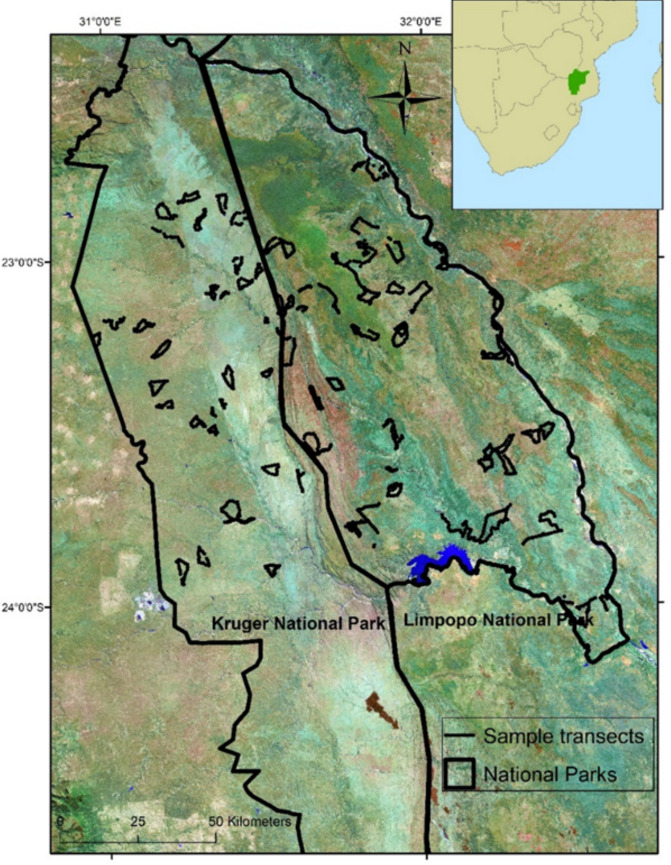
Map of study area showing locations of spoor sampling transects across Kruger National Park, South Africa and Limpopo National Park, Mozambique, within the Greater Limpopo Conservation Area (shown in inset)

We collected detection/non-detection spoor data of lions and other sympatric large carnivores, their collective prey species, and the anthropogenic threats of poaching and pastoralism across northern Kruger and the whole of Limpopo (Fig. 1). We defined sites as 2-km stretches of trail where occasion 1 was the first 1 km and occasion 2 was the second 1 km. In an effort to address positive spatial autocorrelation of detections we included a detection covariate that is either a 1 or 0, where 0 if the animal was not seen in occasion 1 and a 1 if the animal was seen in occasion 1. We made the following assumptions for the estimator *ψ* to be interpreted as probability of site use: (1) sites are closed to changes in occupancy, (2) species are not falsely identified, (3) heterogeneity in occupancy or detection probability are modeled using covariates (MacKenzie et al. 2006). The closure assumption was met by conducting the two sampling occasions of each site immediately one after the other. All tracks recorded were unambiguously identified by KTE, an experienced tracker following Stuart and Stuart (2013) for reference. The sampling scale corresponds with Johnson’s (1980) third-order habitat use scale, reflecting an animal’s short-term habitat use, and the scale at which we were interested, while also being large enough to obtain sufficient detections in low density landscapes. Due to a lack of road networks throughout much of Limpopo, transects were surveyed on foot in both areas. The relative substrate quality for spoor tracking was recorded as a detection covariate on a scale of 1–3 for each 1 km sample, with 1 representing substrate offering the highest probability of spoor detection (e.g., fine sand or shallow mud) and 3 representing the lowest (e.g., grass or hard mud). Only relatively recent, less than approximately 14 days old, spoor was considered as a detection, with the age of tracks and sign estimated by the level of degradation, in consideration of recent weather, and again based on substantial tracking experience. All detection/non-detection and covariate data and transect position were recorded in a Cyber tracker V3.440 program (www.cybertracker.org/).

We built single-species single-season occupancy models for each carnivore species using the covariates expected to influence their occurrence. These covariates included occupancy of each carnivore’s principal prey, direct anthropogenic threats including the occupancy of poaching and pastoralism, indirect threats including distance from settlements, distance to protected area core and abiotic landscape variables including distance from water and landscape type (see Table S.1 for descriptions of covariates for carnivore models). Principal prey for lion and spotted hyena included buffalo, *Syncerus caffer*, and kudu, *Tragelaphus strepsiceros,* for leopard was restricted to impala, *Aepyceros melampus,* and for cheetah and African wild dog included both kudu and impala (Funston et al. 2001; Hayward et al. 2007; Balme et al. 2019; Forbes et al. 2024). The occupancy of prey species, poaching and cattle (pastoralism) were estimated for each site by first building single-species single-season occupancy models for each of these variables (see Table S.1 for descriptions of covariates used for these models) as per Everatt et al. (2014) and Duangchatrasiri et al. (2019). Landscape variables were extracted from the Imagine Image raster "GLTF Landcov" (peackparks.co.za) in ArcGIS 10.1 (www.esri.com). Continuous values were standardized using a z-scale to have a mean of 0 and standard deviation of 1. Collinearity between variables was tested using a Pearson correlation coefficient with an exclusion criterion of *r* > 0.6 in MS Excel v. 1906. Site occupancy (*ψ*) and detection probability (*p*) were estimated using maximum likelihood occupancy functions (MacKenzie et al. 2006) in the single-season single-species option in the R (R Core Team, 2017) package, ‘RPresence’ (Version: 2.12.20; MacKenzie and Hines 2018). Models were ranked based on the Akaike Information Criterion (AIC) and a candidate set was considered for all models with ΔAIC < 2. We ran univariate models because the goal was to investigate carnivore co-occurrence and single variables perform better in two-species models (Richmond et al. 2010).

We then modeled species’ statistical interactions, assuming lions to be the dominant predator in each two-species interaction and used the single-season two-species occupancy estimation function, occMod() with the default option (estimate parameters for the subordinate species conditional on the parameters for the dominant species) in the R package ‘RPresence.’

(Version: 2.12.20; MacKenzie and Hines 2018). We estimated the occupancy parameters; ψA (Probability that the site is occupied by species A, lion), ψBA (Probability that the site is occupied by species B, given species A is present), ψBa (Probability that the site is occupied by species B given species A is absent). We modeled ψA, ψBA, and ψBa incorporating the highest-ranking covariates from the single-species models to account for each species-specific habitat relationships. We also incorporated substrate quality as a detection covariate in all species co-occurrence models. We built the models to test if the occupancy of species B was influenced by species A (ψBa ≠ ψBA) or was independent of species A (ψBa = ψBA).

The species interaction factor (SIF) φ was then calculated using the following formula:

$$\varphi = \frac{\psi AB}{{(\psi BA * \psi Ba)}}$$

Values of φ < 1 indicate avoidance of species A by species B (the two species co-occur less frequently than expected under a hypothesis of independence), values of φ > 1 indicate co-occurrence of the species A and B (the two species co-occur more frequently than expected under a hypothesis of independence) and φ = 1 indicates the two species occur independently (MacKenzie et al. 2004; Richmond et al. 2010; Nagy-Reis et al. 2017).

## Results

Between February and October 2015 we sampled 768, 2 km long sites, including 269 sites in Kruger and 499 sites in Limpopo. Each site was split into two consecutive 1 km sampling occasions, with the two occasions sampled immediately one after the other. The presence of lion was recorded at 96 sites, cheetah at 22 sites, leopard at 114 sites, spotted hyena at 296 sites, and African wild dog at 19 sites (Table S.2).

The average estimated site occupancy for impala was $\overline{\hat{\psi }}$ = 0.540 (range 0.215–0.934), for buffalo was $\overline{\hat{\psi }}$= 0.643 (range 0.002–1.00), and for kudu was $\overline{\hat{\psi }}$= 0.687 (range 0.425–0.925). The average estimated occupancy of bushmeat poaching was $\overline{\hat{\psi }}$ = 0.176 (range 0.006–0.440) and pastoralism was $\overline{\hat{\psi }}$ = 0.198 (range 0.00–0.793). The model selection procedures for prey species and anthropogenic threats are summarized in Table S.3.

### Single-season single-species occupancy models

The detection covariates “substrate” and “p recap,” the probability of recapture covariate, which measured the probability of detection given detection on the previous sample, ranked higher than the null detection model for all carnivores. The probability of recapture covariate had a non-significant but negative association with lion detections in Kruger (*β* = −0.560 ± 0.530) and Limpopo (*β* = −2.086 ± 1.121). In contrast, there was a significant positive relationship with probability of recaptures for the detection of leopard in Kruger (*β* = 0.961 ± 0.441) and Limpopo (*β* = 1.0581 ± 0.517), for cheetah in Limpopo (*β* = 2.852 ± 0.8902 (from 3rd model), African wild dog in Kruger (*β* = 1.791 ± 0.766), and spotted hyena in Kruger (*β* = 0.075 ± 0.328) and Limpopo (*β* = 0.997 ± 0.253). The single-species models with covariates for cheetah in Kruger and African wild dog in Limpopo did not converge due to a low number of detections. Therefore, for these species, we used the null model (Ψ(.)p(.); i.e., no covariates) in the subsequent two-species models, instead of including the covariates from the top model as we did for all other species. Detection probabilities for all others ranged from 0.670 for spotted hyena in Kruger to 0.081 for African wild dog in Kruger, while occupancy estimates ranged from 0.687 for spotted hyena in Kruger to 0.038 for cheetah in Kruger (Table S.2).

The occupancy of lion was best predicted by a positive significant association with availability of water ($\beta =0.247\pm 0.111$) in Kruger and a positive significant association with buffalo occurrence $(\beta =3.308\pm 0.876$) in Limpopo. The relationship between leopard in Kruger and their predictive covariates was less clear, but included non-significant positive relationships with availability of water (*β* = 0.171 ± 0.141), a non-significant negative relationship with proximity to settlements (*β* = −0.205 ± 0.169) and a non-significant positive relationship with the occurrence of kudu (*β* = 2.100 ± 1.700) among the strongest predictors. In contrast, leopard in Limpopo were predicted by a significant positive association with the occurrence of impala ($\beta =18.869\pm 6.326$). Cheetah in Limpopo was predicted by a significant positive association with the occurrence of impala (*β* = 5.297 ± 1.884), a non-significant positive association with distance from settlements (*β* = 1.709 ± 1.048), and significant negative association with the occurrence of cattle (*β* = −3.774 ± 1.548). African wild dog occupancy in Kruger was predicted by a non-significant negative association with the habitat feature bushlands (*β* = −5.493 ± 2.367). Spotted hyena occupancy was best predicted by a significant negative association with bushlands (*β* = −0.718 ± 0.252) in Kruger and a strong positive significant association with kudu (*β* = 37.525 ± 7.968) in Limpopo (Table S.4).

### Two-species occupancy models

The highest-ranking models for each unique lion-carnivore pair, other than lion-spotted hyena in Kruger, were the models predicting that co-occurrence among species was non-independent; that is the occupancy of species B (cheetah, leopard or African wild dog) when species A (lion) was present differed from the occupancy of species B when species A was absent (ψBa ≠ ψBA). Lion-spotted hyena in Kruger, in contrast, displayed a ψBa similar to ψBA indicating near independence; that spotted hyena occupancy was not influenced by lion occupancy (Table 2).

**Table 2:** Occupancy (Ψ), detection probabilities (*p* and *r*), and species interaction factors $(\overline{\hat{\psi }})$ with standard errors, estimated from the co-occurrence models between lions (Species A), assumed to be the dominant species, and each of the syntopic subordinate predators (species B); cheetah, leopard, spotted hyena, and African wild dog, from each park separately.

Species pair	ΨA	ΨBA	ΨBa	rA	pA	pB	rBA	rBa	$(\overline{\hat{\psi }})$
Kruger									
Lion–Cheetah	0.18 (0.15)	0.08 (0.03)	0.01 (0.00)	0.32 (0.25)	0.32 (0.25)	0.35 (0.27)	0.35 (0.28)	0.35 (0.27)	2.75 (0.85)*
Lion–Leopard	0.19 (0.15)	0.36 (0.23)	0.22 (0.13)	0.31 (0.25)	0.31 (0.25)	0.23 (0.19)	0.22 (0.19)	0.23 (0.19)	1.41 (0.23)*
Lion–Spotted hyena	0.19 (0.15)	0.37 (0.32)	0.36 (0.32)	0.31 (0.25)	0.31 (0.25)	0.36 (0.32)	0.36 (0.32)	0.36 (0.32)	1.01 (0.05)*
Lion–African wild dog	0.19 (0.15)	0.26 (0.24)	0.52 (0.37)	0.31 (0.25)	0.31 (0.25)	0.07 (0.02)	0.07 (0.02)	0.07 (0.02)	0.43 (0.41)
Limpopo									
Lion–Cheetah	0.09 (0.09)	0.08 (0.05)	0.04 (0.02)	0.19 (0.16)	0.19 (0.16)	0.27 (0.15)	0.27 (0.14)	0.27 (0.15)	8.07 (10.11)
Lion–Leopard	0.08 (0.08)	0.66 (0.52)	0.34 (0.35)	0.20 (0.17)	0.20 (0.17)	0.06 (0.05)	0.06 (0.05)	0.06 (0.05)	1.42 (0.90)
Lion–Spotted hyena	0.08 (0.08)	0.55 (0.38)	0.24 (0.26)	0.20 (0.17)	0.20 (0.17)	0.23 (0.20)	0.23 (0.20)	0.23 (0.20)	2.85 (2.25)
Lion–African wild dog	0.08 (0.08)	0.03 (0.00)	0.01 (0.00)	0.20 (0.16)	0.20 (0.16)	0.39 (0.34)	0.38 (0.34)	0.39 (0.34)	11.22 (14.4)

Ψ*A*: Probability of occupancy species A

Ψ*BA*: Probability of occupancy species B, given that species A is present

Ψ*Ba*: Probability of occupancy species B, given that species A is absent

*rA*: Probability of detection species A, given that both are present

*pA*: Probability of detection species A, given species B is absent

*pB*: Probability of detection species B, given species A is absent

*rBA*: Probability of detection species B, given that both are present and species A was detected

*rBa*: Probability of detection species B, given that both are present and species A was not detected

*95% significance

**Fig. 2:**
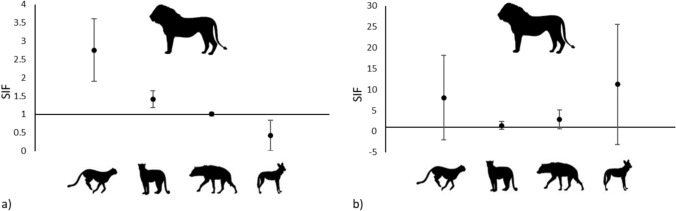
Species interactions factors (SIF) (± 95% CI), indicating co-occurrence, between lions and each of the syntopic subordinate predators (from left to right, cheetah, leopard, spotted hyena, and African wild dog) averaged across **a** well-protected Kruger National Park and **b** nominally protected Limpopo National Park. Species interaction values of 1 (horizontal line) indicate lions and syntopic subordinate predators occur independently, values of < 1 indicate negative association (avoidance of lion areas by subordinate predators), and values of > 1 indicate positive association, co-occurrence between species pair (selection of habitats where lions co-occur). Values where CI do not overlap 1 are considered significant

Species interaction factors (SIF) varied among species pairs and among the same pair combinations between sites. Specifically, the average species interaction factor from the top models indicated a positive, significant, association between lions and cheetah in Kruger (φ = 2.75 ± 0.85) and a positive, non-significant, association in Limpopo (φ = 8.07 ± 10.11). Thus, the top models showed that cheetah occupied 8% of sites where lions were present and 1% of sites where lions were absent across Kruger, and 8% of sites where lions were present and 4% of sites where lions were absent across Limpopo. The average species interaction factors for lions and leopards were positive and significant in Kruger (φ = 1.41 ± 0.23) and positive, although non-significant, in Limpopo (φ = 1.42 ± 0.90). Leopards occupied an average of 36% of sites where lions were present and 22% of sites where lions were absent in Kruger and 66% of sites where lions were present and 44% of sites where lions were absent in Limpopo. There was a significant near-independent association between lions and spotted hyenas in Kruger (φ = 1.01 ± 0.05) and a positive, although non-significant, association in Limpopo (φ = 2.85 ± 2.25). Spotted hyenas occupied an average of 37% of sites where lions were present and 36% of sites where lions were absent in Kruger and 55% of sites where lions were present and 24% of sites where lions were absent in Limpopo. There was a non-significant slight negative association between lions and African wild dogs in Kruger (φ = 0.43 ± 0.41), and a non-significant positive association in Limpopo (φ = 11.22 ± 14.38). Nonetheless, African wild dogs occupied an average of 26% of sites where lions were present and 52% where lions were absent in Kruger, and 3% of sites where lions were present and 1% of sites where lions were absent in Limpopo (Table 2, Fig. 2).

All competitive models (∆AIC < 2) describing site-specific species co-occurrence in Kruger included the effects of water, SP (no conditional effect of species A on species B) and INT (conditional effects of species A on species B). The models describing lion-spotted hyena and lion-African wild dog associations also included bushlands, and models describing lion-leopard associations also included kudu, settlements, impala, grasslands, and thickets (Table 3).

**Table 3:** Summary of top-ranking species co-occurrence models (∆AIC < 2) describing site use of lion with cheetah leopard, spotted hyena, and African wild dog across Kruger and Limpopo National Parks

Species pair	Models	AIC	∆AIC	*w*	Model Likelihood	K	*-*2 l
Kruger							
Lion–Cheetah	Ψ(Water, None, SP + INT)p(Substrate)	525.48	0.00	0.58	1.00	10	505.48
Lion–Leopard	Ψ(Water, None, SP + INT),p(Substrate)K	881.73	0	0.07	1	10	861.73
	Ψ(Water, Kudu, SP + INT),p(Substrate)K	881.76	0.03	0.07	0.98	11	859.76
	Ψ(Water, None, SP),p(Substrate)K	881.85	0.12	0.07	0.94	9	863.85
	Ψ(Water, Kudu, SP),p(Substrate)K	882.24	0.51	0.05	0.77	10	862.24
	Ψ(Water, Settlement, SP),p(Substrate)K	882.61	0.88	0.05	0.65	10	862.61
	Ψ(Water, Settlement, SP + INT),p(Substrate)K	882.67	0.94	0.04	0.63	11	860.67
	Ψ(Water, Impala, SP + INT),p(Substrate)K	882.73	1	0.04	0.61	11	860.73
	Ψ(Water, Grasslands, SP + INT),p(Substrate)K	882.93	1.2	0.04	0.55	11	860.93
	Ψ(Water, Water, SP + INT),p(Substrate)K	883.05	1.32	0.04	0.52	11	861.05
	Ψ(Water, Bushlands, SP),p(Substrate)K	883.31	1.58	0.03	0.45	10	863.31
	Ψ(Water, Thicket, SP + INT),p(Substrate)K	883.35	1.62	0.03	0.45	11	861.35
	Ψ(Water, Bushlands, SP + INT),p(Substrate)K	883.51	1.78	0.03	0.41	11	861.51
	Ψ(Water, Impala, SP)p(Substrate)K	883.52	1.79	0.03	0.41	10	863.52
Lion–Spotted hyena	Ψ(Water, Bushlands, SP)p(Substrate)	1134.65	0.00	0.40	1.00	10	1114.65
	Ψ(Water, Bushlands, SP + INT)p(Substrate)	1135.49	0.84	0.26	0.66	11	1113.49
Lion–African wild dog	Ψ(Water, Bushlands, SP)p(Substrate)	601.61	0.00	0.40	1.00	10	581.61
	Ψ(Water, Bushlands, SP + INT)p(Substrate)	603.34	1.72	0.17	0.42	11	581.34
Limpopo							
Lion–Cheetah	Ψ(Buffalo, Impala, SP)p(Substrate)	412.69	0	0.30	1	10	392.69
Lion–Leopard	Ψ(Buffalo, Impala, SP + INT)p(Substrate)	632.26	0	0.64	1	11	610.26
Lion-Spotted hyena	Ψ(Buffalo, Kudu, SP + INT)p(Substrate)	1017.37	0.00	0.47	1.00	11	995.37
	Ψ(Buffalo, Kudu, SP)p(Substrate)	1019.13	1.76	0.19	0.41	10	999.13
Lion-African wild dog	Ψ(Buffalo, None, SP)p(Substrate)	296.03	0.00	0.35	1.00	9	278.03
	Ψ(Buffalo, None, SP + INT)p(Substrate)	296.22	0.18	0.32	0.91	10	276.22

SP estimates a different occupancy for each species with no conditional effects, (where Ψ BA = Ψ Ba) and INT estimates occupancy of each species with conditional effect of species A on species B, (Ψ A, Ψ BA, and Ψ Ba)

All competitive models describing site-specific species co-occurrence in Limpopo included the variables buffalo, SP, and INT. Variables influencing lion-leopard and lion-cheetah associations also included impala and variables influencing lion-spotted hyena associations also included kudu (Table 3).

**Fig. 3:**
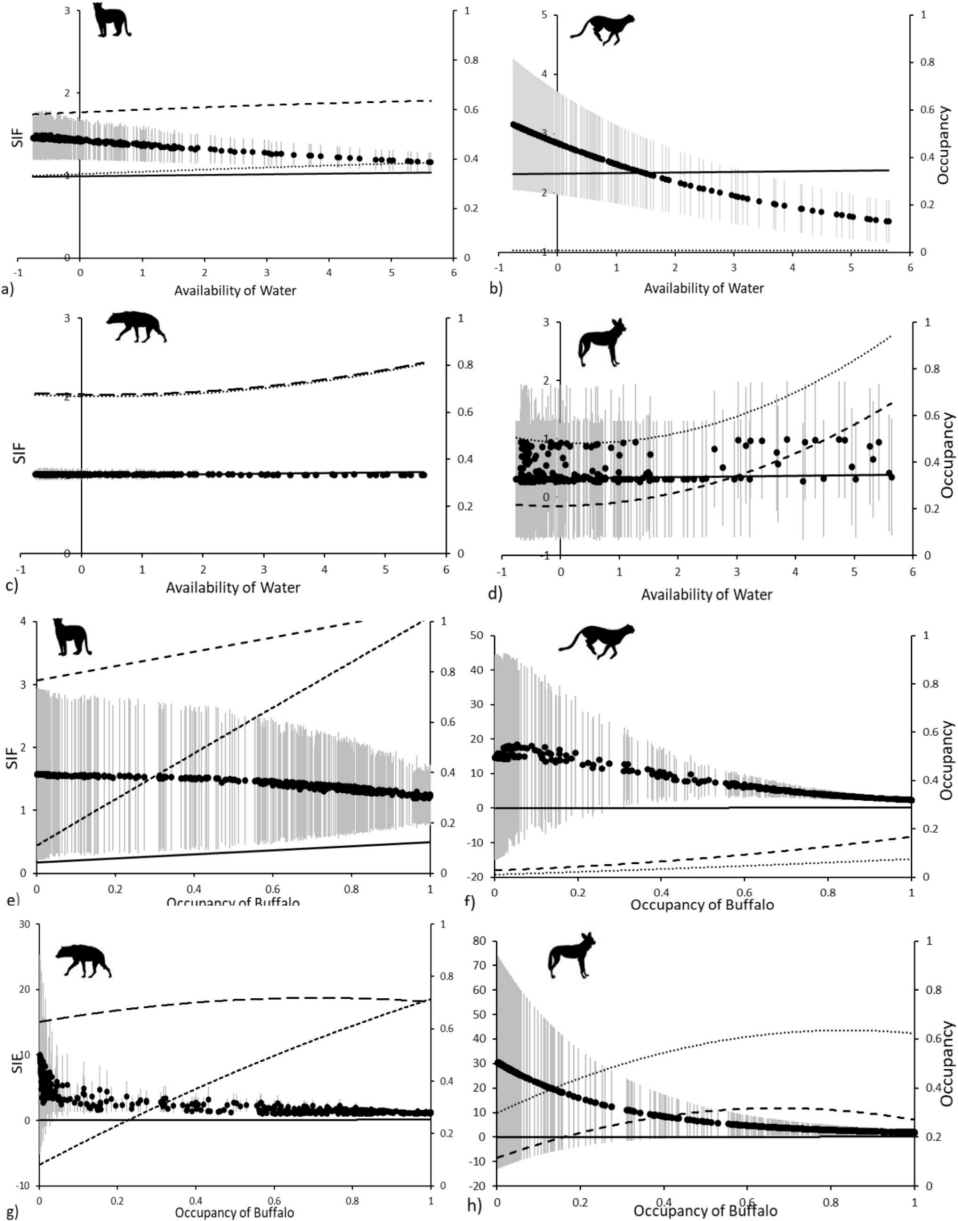
Species Interaction Factors (SIF) (solid black dots with SE in gray), indicating co-occurrence, between lions and leopards (**a** and **e**), lions and cheetahs (**b** and **f**), lions and spotted hyenas (**d** and **g**) and lions and African wild dogs (**e** and **h**), conditional on the top-ranking variables for species pairs co-occurrence including the relative availability of water for species pairs in Kruger (**a**, **b**, **c** & **d**) and the occupancy of buffalo for species pairs in Limpopo (**e**, **f**, **g** and **h**), shown with the occupancy probability of each carnivore relative to the presence of lions (dashed lines) or absence of lions (dotted lines) and the occupancy of lions (solid line). Species interaction factors and conditional occupancy estimates were obtained from two-species occupancy models and lion occupancy from single-species occupancy models developed from spoor data collected across Kruger and Limpopo National Parks

The relative availability of water had a negative effect on lion and leopard and on lion and cheetah associations in Kruger with pair-wise species interaction factors ranging from 1.456 ± 0.262 and decreasing to 1.163 ± 0.115 for lion-leopard and 3.156 ± 1.101 decreasing to 1.521 ± 0.357 for lion-cheetah at sites with the lowest to highest water availability. The same variable had little effect on lion and spotted hyena associations with species interaction factors ranging from 1.009 ± 0.0579 to 1.004 ± 0.024 and had a slight positive influence on lion-African wild dog associations with species interaction factors ranging from 0.309 ± 0.453 increasing to 0.346 ± 0.448 at sites with the lowest to highest water availability (Fig. 3). Pair-wise species associations between lions and African wild dogs in Kruger declined with the relative availability of bushlands with species interaction factors ranging from 0.991 ± 0.0458 to 0.307 ± 0.454. Regardless of the relative availability of bushlands, conditional occupancy of spotted hyenas was unaffected by lion occupancy with species interaction factors ranging from 1.002 ± 0.012 to 1.012 ± 0.077 at sites with the lowest to highest bushlands availability.

The occupancy of buffalo had a negative effect on all species pair associations in Limpopo with pair-wise species interaction factors ranging from 1.572 ± 1.364 to 1.227 ± 0.443 for lion-leopard and 14.632 ± 29.832 to 2.365 ± 0.675 for lion-cheetah, 10.083 ± 15.209 to 1.156 ± 0.147 for lion-spotted hyena and 30.371 ± 43.320 to 1.917 ± 1.102 for lion-African wild dog at sites with the lowest to highest buffalo occurrence (Fig. 3). The occupancy of impala had a slight negative effect on lion-leopard associations in Limpopo with species interaction factors ranging from 1.572 ± 1.364 to 1.195 ± 0.391, and a non-significant large negative effect on lion-cheetah associations with species interaction factors ranging from 14.632 ± 29.832 to 2.608 ± 0.776, at sites with the lowest to highest impala occurrence. The occupancy of kudu had a negative effect on lion-spotted hyena associations in Limpopo with pair-wise species interaction factors ranging from 10.083 ± 15.209 to 1.056 ± 0.069 at sites with the lowest to highest kudu occurrence.

## Discussion

Here we employed occupancy models to investigate species-habitat relationships for each of five larger syntopic carnivores occurring across two National Parks and a gradient of anthropogenic impacts. We then built multi-species occupancy models to provide insights of the drivers of each species’ occurrence across the landscape, in response to lion occurrence and investigate the contributions of variables influencing these intra-guild interactions. By including the top predictor of occupancy for each species within the two-species occupancy modeling procedure, we were able to reduce bias associated with any confounded species-habitat associations (Richmond et al. 2010; Nagy-Reis et al. 2017; Ladle et al. 2018).

### Single-species habitat associations

Our single-species single-season occupancy models revealed that the occupancy of all carnivore species across both parks was closely related to the occurrence of their prey or to landscape features related to access to their prey. While the specific variables predicting carnivore occupancy differed between Kruger and Limpopo; the differences likely reflected variations in the relative availability of prey between the two parks. For instance, prey densities in Limpopo are far below ecological carrying capacity (Baghai et al. 2018) and most species are distributed heterogeneously across the landscape (Everatt et al. 2014). The relative scarcity and patchy distribution of prey in Limpopo therefore makes prey occurrence itself a direct limiting factor for carnivore occupancy. In Kruger by contrast, prey densities are close to their ecological carrying capacity (Baghai et al. 2018) and as such the relative occurrence of prey would be less of a limiting factor for carnivores. Instead in Kruger we found carnivore occupancy is better predicted by landscape features that are themselves known to be selected for by important prey species or by features which may facilitate the capture of prey (Mosser et al. 2009). For instance, lions in Limpopo showed a selection for sites with buffalo (model likelihood 1) while in Kruger they showed an equally strong selection for habitats near water; a habitat feature selected for by buffalo (Everatt 2020). The dense vegetation found along riparian areas, which is captured by the water variable also provides optimal ambush conditions for lions (Kittle et al. 2016), thus improving prey catchability. A similar pattern can be observed with spotted hyenas which, in Limpopo, showed a selection (model likelihood 1) for sites with kudu, while in Kruger showed an equally strong selection for bushlands, a habitat type frequented by kudu (Everatt 2020). Interestingly, in addition to the above-mentioned spatial responses by carnivores to the availability of prey, Forbes et al (2024) examined lion and leopard diet in Limpopo and Kruger and found that while carnivores selected for preferred prey species across both parks, they also increased their dietary breath to include more smaller prey species in the prey depleted Limpopo.

### Interactions between lions and subordinate carnivores

We found significant species co-occurrence between lion and cheetah pairs, lion and leopard pairs, and lion and spotted hyena pairs in Kruger. The larger standard errors calculated from the other dyadic associations are likely due to the limited data available caused by the lower species occupancies in Limpopo generally, and the lower occupancy of African wild dogs in both parks. The nature of these co-occurrence dynamics provided limited support for the competitive exclusion hypothesis and more support for the co-occurrence of most species’ pairs, with the exception of lion-African wild dog interactions.

African wild dogs in Kruger showed avoidance of lions (Fig. 1) and their occupancy was higher given lion absence (Fig. 3). This suppression of African wild dogs by lions at this third order of spatial/habitat scale agrees with previous findings of African wild dog avoidance of lions (Dröge et al. 2017; Goodheart et al. 2024; and including Strampelli et al. 2023 who employed a similar study design as used here), and supports the negative associations found between African wild dog recruitment and increased lion densities (Groom et al. 2017). These negative associations found between lions and African wild dogs suggests a level of proactive (coarse scale) (Broekhuis et al. 2013) avoidance of lions. African wild dogs are known to suffer high levels of kleptoparasitism and harassment by lions and have been shown to forfeit access to resource-rich but lion-occupied habitats at a much larger spatial scale (Mills and Mills 2017; Marnewick et al. 2019; Goodheart et al. 2024), even relocating den sites in avoidance of lions (Swanson et al. 2014). Furthermore, we found that co-occurrence between lions and African wild dogs continued to decline with a corresponding increase in lion occupancy.

In contrast, we found that lions and cheetahs showed strong positive co-occurrence patterns in Kruger, with cheetah occupancy 8 × higher at sites with lions than at sites without lions. Lions and leopards showed slight positive co-occurrence in Kruger, and lions and spotted hyenas showed close to independence in their interactions in Kruger. Less significant results further suggest that leopards showed slight positive associations with lions in Limpopo (Table 2; Fig. 2). Further evidence of co-occurrence within the guild was also found in that the probability of occupancy of leopard and cheetah given the presence of lions was higher than these species’ probability of occupancy given the absence of lions (Fig. 3).

The selection of sites with lions by cheetah and leopard may seem counter-intuitive, as both species are predated on by lions (Balme et al. 2017; Mills and Mills 2017). However, observational studies have documented cheetah only abandoning sites when lions approached to within 60 m in the Kagaldi National Park, South Africa (Mills and Mills 2017) and leopards to only abandon a path of travel once lions were within 50 m in Sabi Sands Game Reserve, South Africa (Balme et al. 2017). The positive species’ interactions found by this study at the 2 km spatial scale, contrasting with the above published negative interactions at much smaller scales may simply indicate that leopards and cheetah exhibit a reactive (fine scale) response to lions. A reactive response to lions by leopards and cheetah has also been documented in other systems (Broekhuis et al. 2013; Strampelli et al. 2023), including by cheetah to the reintroduction of lions (Cornhill et al. 2022).

The positive lion-cheetah and lion-leopard co-occurrence found here therefore suggest the possibility that these two sub-ordinant predators recognize the presence of lions as an indicator of habitat quality; at least at the predator densities found in these parks.

Theoretically, higher densities of either lions or sympatric carnivores would lead to higher probabilities of intra-guild encounters and therefore competition. This also was supported here where interactions between lions and leopards in Kruger, and between lions and cheetahs in both Kruger and Limpopo, although positive, all declined with corresponding increases in lion occupancies (Fig. 3). This trend may indicate the subtle effects of increasing interference competition, avoidance of lions, which is otherwise largely moderated by the combination of adequate prey in Kruger and refugia habitat in both parks.

The near-independent associations found between lions and spotted hyenas found here (Fig. 2) may reflect these species’ known dynamic competitive relationships. Lions and spotted hyenas are known to kleptoparasitize and kill one another, with the outcome of this interaction largely based on relative group size and composition (Lehmann et al. 2017). Furthermore, and unlike lion-leopard and lion-cheetah associations, co-occurrence between lions and spotted hyenas changed little across sites irrespective of lion occupancy (Fig. 3).

Together these trends support the hypothesis that the functional effects of lions on some, but not all, syntopic carnivores are dictated by the relative abundance of lions. It is therefore probable that other functional effects of lions, including pan-trophic interactions, may also be dependent on minimum lion occurrences or densities. While there are few documented cases of trophic cascades resulting from changes in lion abundance (Tossens et al. 2024; but see Brashares et al. 2010), it may non the less be an important ecological consideration, particularly where lions occur in less productive systems with lower intra-guild redundancy and are thus likely to have stronger functional effects. Lions across Africa have undergone significant population and range contractions over the last decades (Nicholson et al. 2023), and may no longer exist at functionally effective densities across much of their remaining range. The effect of lions on African wild dogs is the possible exception, where this study, as well as Goodheart et al (2024), found this relationship to only require a minimum density of lions.

### Effects of resource availability on species interactions

The top-ranking variables influencing species pairs’ co-occurrence were all reflective of resource, water and prey, availability (Table 3). We also observed general trends where increasing resource (water) availability in Kruger led to decreasing lion-leopard and lion-cheetah co-occurrence. All species dyadic associations decreased slightly, indicating increased avoidance of lions, with increasing resource (buffalo) availability in Limpopo (Fig. 3). These trends may reflect a process whereby greater availability of resources, increases available niche, permitting greater spatial avoidance and thus reducing competition within the guild (Lamichhane et al. 2019; Shao et al. 2021). These results therefore provided evidence in support of our final hypothesis that co-occurrence in carnivore communities is, largely, positively related to resource availability (Périquet et al. 2015; Shao et al. 2021).

## Conclusion

As wildlife communities become increasingly impacted by human pressures it is important to understand the mechanisms influencing species co-occurrence. By employing two-species occupancy models to investigate the functional effect of African lions on the presence of co-occurring large carnivores, this study found that, while each species’ occupancy was best predicted by access to their own key resources, co-occurrence between all species pairs was influenced by the relative availability of resources, with increasing avoidance of lions by cheetah, leopard, and spotted hyena as resources became more abundant. However, the strength of associations differed between pairs, with a competitive exclusion hypothesis only explaining co-occurrence between lions and African wild dogs. Furthermore, this study found that avoidance of lions by co-occurring carnivores increased with increased lion occupancy. These findings will be useful for predicting the impacts of changes in resources and changes in the relative densities of apex carnivores on carnivore community dynamics, information pertinent to conservation area management and particularly to the re-wildling of carnivore communities.

## Supplementary Information

Below is the link to the electronic supplementary material.

Supplementary file1 (DOCX 30 KB)

## Data Availability

Data are available from corresponding author on request.

## Code availability

Codes are available from corresponding author on request.
