# Supplementary material for: The functional effects of African lions on co-occurring carnivores differ across species pairs and with changes in resource availability and lion abundance
Source: Oecologia. 2026 Jan 8;208(2):21. doi: 10.1007/s00442-025-05855-5 (PMC12783162; doi:10.1007/s00442-025-05855-5)
Supplement: Supplementary file 1 — Supplementary file1 (DOCX 30 KB) [file 442_2025_5855_MOESM1_ESM.docx]

**The functional effects of African lions on co-occurring carnivores differs across species pairs and with changes in resource availability and lion abundance.**

Kristoffer T. Everatt ^a b^, Leah Andresen^a^, Jennifer F. Moore ^c^, Jim Hines ^d^, Graham I.H. Kerley ^a^

^a^Centre for African Conservation Ecology, Department of Zoology, Nelson Mandela University, Gqeberha 6031, South Africa

^b^Panthera, New York, NY, USA

^c^ Moore Ecological Analysis and Management, LLC, St. Petersburg, FL, USA

^d^ USGS, Eastern Ecological Science Center, Laurel, MD, USA

**Table S.1.** Independent variables expected to describe impala, kudu, buffalo, cattle and bushmeat poacher habitat use, their assumed biological relevance, metric recorded and data source

| **Independent variable** | **Biological relevance to herbivore occurrence** | **Metric recorded** | **Source** |
| --- | --- | --- | --- |
| Poaching (presence) | Direct persecution | 0/1 | Spoor surveys |
| Settlements/agriculture (distance from) | Direct persecution | Euclidean distance from settlement pixels | Landcover raster |
| Protected areas (distance from) | Higher protection in Kruger NP | Euclidean distance from edge of Kruger | Landcover raster |
| Water (distance from) | Habitat requirement | Euclidean distance from water pixels | Landcover raster |
| Pans | Important foraging habitat | Number of pixels overlapping point | Landcover raster |
| Grasslands | Important foraging habitat | Number of pixels overlapping point | Landcover raster |
| Forests | Shelter | Number of pixels overlapping point | Landcover raster |
| Bushlands | Forage and shelter | Number of pixels overlapping point | Landcover raster |
| Thickets | Forage and shelter | Number of pixels overlapping point | Landcover raster |

Land cover rasters obtained from Imagine Image raster “GLTF Landcov” projected in WGS_1984_UTM_Zone_36S (www.peaceparks.co.za)

**Table S.2**. Number of 2-km sites where each species was detected (*d*), naïve occupancy of each species (*N*), probability of occupancy $( \bar{\hat{\psi}}$) and probability of detection$( \bar{\hat{p}}$) at the 2 km spatial scale for species in Kruger and Limpopo national parks from model averaging of top-ranking single-season single-species occupancy models.

|  | *d* | *N* | $\bar{\hat{\psi}}$(SE) | $\bar{\hat{p}}$ (SE) |
| --- | --- | --- | --- | --- |
| Lion - Kruger | 67 | 0.25 | 0.312 (0.070) | 0.569 (0.116) |
| Lion - Limpopo | 29 | 0.06 | 0.096 (0.066) | 0.486 (0.256) |
| Leopard - Kruger | 68 | 0.25 | 0.449 (0.141) | 0.361 (0.111) |
| Leopard - Limpopo | 46 | 0.09 | 0.589 (0.315) | 0.089 (0.099) |
| Cheetah - Kruger* | 7 | 0.03 | 0.038 (0.019) | 0.443 (0.206) |
| Cheetah - Limpopo | 15 | 0.03 | 0.184 (0.340) | 0.245 (0.224) |
| African wild dog - Kruger | 16 | 0.06 | 0.550 (0.470) | 0.081 (0.235) |
| African wild dog - Limpopo | 3 | 0.01 | . | . |
| Spotted hyena - Kruger | 163 | 0.61 | 0.687 (0.105) | 0.670 (0.066) |
| Spotted hyena - Limpopo | 133 | 0.27 | 0.622 (0.169) | 0.281 (0.042) |

*Estimated from model: psi(.)p(.)

**Table S.3.** Summary of model selection procedure describing site (2 km) use of impala, buffalo, kudu, poachers and cattle across Kruger and Limpopo national parks

| **Models** | **∆AIC** | ***w*** | **K** |
| --- | --- | --- | --- |
| Impala |  |  |  |
| Ψ(Settlement + Protected area),p(Substrate) | 0.00 | 0.4930 | 5 |
| Ψ(Settlement + Protected area + Water),p(Substrate) | 1.54 | 0.2282 | 6 |
| Ψ(Settlement + Protected area + Grasslands),p(Substrate) | 1.92 | 0.1887 | 6 |
| Ψ(Settlement + Protected area+ Grasslands + Water),p(Substrate) | 3.40 | 0.0901 | 7 |
| Buffalo |  |  |  |
| Ψ(Cattle + Settlement + Protected area + Grasslands),p(Substrate) | 0.00 | 0.6769 | 7 |
| Ψ(Cattle + Settlement + Protected area),p(Substrate) | 2.39 | 0.2049 | 6 |
| Ψ(Cattle + Settlement + Protected area + Grasslands + Water),p(Substrate) | 3.83 | 0.0997 | 7 |
| Kudu |  |  |  |
| Ψ(Settlement + Protected area + Water),p(Substrate) | 0.00 | 1.000 | 6 |
| Poaching |  |  |  |
| Ψ(Settlement + Protected area + Water + Impala),p(Substrate) | 0.00 | 0.4038 | 6 |
| Ψ(Protected area),p(Substrate) | 0.94 | 0.2524 | 4 |
| Ψ(Settlement + Protected area),p(Substrate) | 2.23 | 0.1324 | 5 |
| Ψ(Protected area + Impala),p(Substrate) | 2.67 | 0.1063 | 5 |
| Ψ(Protected area + Water),p(Substrate) | 2.69 | 0.1052 | 5 |
| Cattle |  |  |  |
| Ψ(Settlement + Protected area + Grass),p(Substrate) | 0.00 | 0.4485 | 6 |
| Ψ(Settlement + Protected area + Water),p(Substrate) | 0.41 | 0.3654 | 6 |
| Ψ(Settlement + Protected area + Grass + Water),p(Substrate) | 1.76 | 0.1860 | 7 |

*∆AIC is the difference in AIC values between each model and the model with the lowest AIC value *w* is the AIC model weight, K is the number of parameters in the model

**Table S.4.** Summary of single-species top-ranking (∆AIC<2) models describing site (2-km) use of lion, leopard, cheetah, African wild dog and spotted hyena across Kruger and Limpopo national parks.

| **Models** | **AIC** | **∆AIC** | ***w*** | **Model Like** | **K** | ***-*2l** |
| --- | --- | --- | --- | --- | --- | --- |
| Lion Kruger |  |  |  |  |  |  |
| Ψ(Water),p(Substrate) | 441.83 | 0 | 0.32 | 1 | 5 | 431.83 |
| Ψ(Water),p(Substrate + P recap) | 442.74 | 0.91 | 0.21 | 0.64 | 6 | 430.74 |
| Lion Limpopo |  |  |  |  |  |  |
| Ψ(Buffalo),p(Substrate + P recap) | 256.05 | 0 | 0.49 | 1 | 6 | 244.05 |
| Ψ(Buffalo),p(Substrate) | 257.43 | 1.39 | 0.24 | 0.50 | 5 | 247.43 |
| Leopard Kruger |  |  |  |  |  |  |
| Ψ(.),p(Substrate) | 441.12 | 0 | 0.14 | 1 | 4 | 433.12 |
| Ψ(Water),p(Substrate) | 441.38 | 0.26 | 0.12 | 0.88 | 5 | 431.38 |
| Ψ(Settlement),p(Substrate) | 441.61 | 0.49 | 0.11 | 0.78 | 5 | 431.61 |
| Ψ(Kudu),p(Substrate) | 441.62 | 0.49 | 0.11 | 0.78 | 5 | 431.62 |
| Ψ(Grasslands),p(Substrate) | 442.66 | 1.54 | 0.07 | 0.46 | 5 | 432.66 |
| Ψ(Water),p(Substrate + P recap) | 442.83 | 1.71 | 0.06 | 0.43 | 6 | 430.83 |
| Ψ(Impala),p(Substrate) | 442.94 | 1.81 | 0.06 | 0.40 | 5 | 432.94 |
| Ψ(Thicket),p(Substrate) | 442.97 | 1.85 | 0.06 | 0.40 | 5 | 432.97 |
| Ψ(.),p(Substrate + P recap) | 443.04 | 1.92 | 0.05 | 0.38 | 5 | 433.04 |
| Ψ(Bushlands),p(Substrate) | 443.08 | 1.96 | 0.05 | 0.38 | 5 | 433.08 |
| Leopard Limpopo |  |  |  |  |  |  |
| Ψ(Impala),p(Substrate + P recap) | 369.31 | 0 | 0.64 | 1 | 6 | 357.31 |
| Ψ(Impala),p(Substrate) | 370.93 | 1.62 | 0.29 | 0.45 | 5 | 360.93 |
| Cheetah Limpopo |  |  |  |  |  |  |
| Ψ(Impala),p(Substrate) | 165.33 | 0 | 0.21 | 1 | 5 | 155.33 |
| Ψ(Cattle),p(Substrate) | 165.47 | 0.15 | 0.19 | 0.93 | 5 | 155.47 |
| Ψ(Impala),p(Substrate + P recap) | 165.85 | 0.52 | 0.16 | 0.77 | 6 | 153.85 |
| Ψ(Settlement),p(Substrate + P recap) | 166.75 | 1.42 | 0.10 | 0.49 | 6 | 154.75 |
| Ψ(Settlement),p(Substrate) | 166.75 | 1.43 | 0.10 | 0.49 | 5 | 156.75 |
| Ψ(Cattle),p(Substrate + P recap) | 166.81 | 1.49 | 0.10 | 0.48 | 6 | 154.81 |
| African wild dog Kruger |  |  |  |  |  |  |
| Ψ(Bushlands),p(Substrate + P recap) | 154.97 | 0 | 0.45 | 1 | 6 | 142.97 |
| Ψ(Settlement),p(Substrate + P recap) | 156.42 | 1.45 | 0.22 | 0.48 | 6 | 144.42 |
| Spotted hyena Kruger |  |  |  |  |  |  |
| Ψ(Bushlands),p(Substrate) | 688.94 | 0 | 0.64 | 1 | 5 | 678.94 |
| Ψ(Bushlands),p(Substrate + P recap) | 690.89 | 1.95 | 0.24 | 0.38 | 6 | 678.89 |
| Spotted hyena Limpopo |  |  |  |  |  |  |
| Ψ(Kudu),p(Substrate + P recap) | 755.90 | 0 | 0.87 | 1 | 6 | 743.90 |

*∆AIC is the difference in AIC values between each model and the model with the lowest AIC value *w* is the AIC model weight, Model Like is the likelihood that this model explains variation in the data, K is the number of parameters in the model, and −2l is twice the negative log-likelihood value.
